# Positivity of Antigen Tests Used for Diagnosis of Lymphatic Filariasis in Individuals Without *Wuchereria bancrofti* Infection But with High *Loa loa* Microfilaremia

**DOI:** 10.4269/ajtmh.16-0547

**Published:** 2016-12-07

**Authors:** Sébastien D. Pion, Céline Montavon, Cédric B. Chesnais, Joseph Kamgno, Samuel Wanji, Amy D. Klion, Thomas B. Nutman, Michel Boussinesq

**Affiliations:** 1 Unité Mixte Internationale 233, Institut de Recherche pour le Développement, Université de Montpellier, Institut National de la Santé et de la Recherche Médicale, Montpellier, France; 2 Centre de Recherche sur les Filarioses et autres Maladies Tropicales, Yaoundé, Cameroon; 3 Faculty of Medicine and Biomedical Sciences, University of Yaoundé 1, Yaoundé, Cameroon; 4 Research Foundation for Tropical Diseases and Environment, Buea, Cameroon; 5 Department of Microbiology and Parasitology, University of Buea, Buea, Cameroon; 6 National Institutes of Health, Bethesda, Maryland

## Abstract

Since the mid-2000s, the immunochromatographic card test (ICT), a point-of-care test for detecting *Wuchereria bancrofti* circulating filarial antigens (CFAs), has been the backbone for mapping and monitoring lymphatic filariasis (LF) worldwide. Recently, there have been instances in which CFA positivity has been associated with *Loa loa* microfilaremia. Here, we examined the association, at both the community and individual levels, between *L. loa* and CFA using additional diagnostic tools (quantitative polymerase chain reaction [qPCR], Og4C3 enzyme-linked immunosorbent assay, and IgG4 antibodies to Wb123 assays) to demonstrate the relationship between *L. loa* microfilaremia and ICT positivity. In May 2013, peripheral blood was collected during the day from 1,812 individuals living in southern Cameroon. ICT tests were done on the spot, and positive individuals were resampled at night. Results of qPCR and Wb123 assays concurred proving the absence of *W. bancrofti* infection. Og4C3 assays indicate a quantitative relationship between the level of *L. loa* microfilaremia and that of CFA. This was confirmed by epidemiological analyses, which reveal a strong association between *L. loa* microfilaremia and ICT positivity, with 50% of ICT reacting to *L. loa* when its microfilarial density exceeds 30,000 microfilariae/mL. At the community level, the proportion of positive ICT would exceed 2% when the prevalence of *L. loa* microfilaremia in the total population is above 20%. This has significant implications in terms of mapping and control of LF caused by *W. bancrofti* in *Loa*-endemic areas. Cross-reactivity of ICT with *L. loa* has to be considered in the context of both individual and community diagnostics.

## Introduction

The immunochromatographic card test (ICT) (Binax Now Filariasis ICT test; Alere Inc., Waltham, MA), a point-of-care test for detecting *Wuchereria bancrofti* circulating filarial antigens (CFAs), has been widely used for mapping and monitoring lymphatic filariasis (LF) worldwide. In recent years, puzzling results have emerged from ICT-based LF mapping surveys conducted in central Africa, raising questions about the validity of LF distribution maps and current estimates of the prevalence of infection in this subregion.^1^,^2^

In practice, variations in the prevalence of antigenemia (CFA) over relatively small distances indicates that the geographical distribution of LF is very uneven, and the infection can be found at very low levels in some communities, possibly below the threshold above which transmission is sustainable. Furthermore, the presence of CFA is not always corroborated by *W. bancrofti* microfilaremia.^3^ Moreover, there have been several instances in which CFA positivity has been associated with *Loa loa* microfilaremia.^4^–^6^ In a study conducted in Orientale Province in eastern Democratic Republic of Congo, an association between *L. loa* microfilarial density assessed at night (between 9:00 pm and 1:00 am) and ICT positivity was observed,^4^ although there was no attempt to demonstrate a quantitative relationship between *L. loa* microfilaremia (during the day) and ICT positivity. In addition, quantitative polymerase chain reaction (qPCR) on dried night blood retrieved from stained smears or ICT card application pads confirmed the presence of *L. loa* and the absence of *W. bancrofti* DNA.^4^

Another study, performed in three different regions of Cameroon, demonstrated that the rates of ICT positivity (ranging from 0% to 11.1% across 42 villages surveyed) were positively correlated with the prevalence of *L. loa* microfilaremia (Pearson's *r* = 0.53, *P* = 0.0003).^5^ At the individual level, the probability of ICT positivity gradually and significantly increased with the diurnal *L. loa* microfilarial density. No molecular analyses were performed during this study and, because *L. loa* and *W. bancrofti* microfilariae (mf) are of similar size and shape, the presence of small numbers of *W. bancrofti* mf could have been missed when *L. loa* mf density was high in night blood samples.^5^

The original objective of the present work was to assess the levels of *W. bancrofti* and *L. loa* infection in a population previously reported to be co-endemic for the two filarial parasites and to analyze the pattern of association between these two filarial parasites at both the community and individual levels. To achieve this objective, populations of 26 communities in southern Cameroon were screened using standard techniques: ICT and nighttime thick blood smears for *W. bancrofti* and daytime thick blood smears for *L. loa*. Despite a significant number of positive ICT tests, not a single *W. bancrofti* mf was identified in the nighttime blood smears. To investigate this further, additional follow-up samples were collected for examination by qPCR and for detection of antibodies to the highly specific *W. bancrofti* specific antigen, Wb123,^7^–^9^ and individual- and community-level analyses were performed to determine the relationships between *L. loa* infection levels and rates of ICT positivity.

## Materials and Methods

### Study site.

The study area included 26 communities located within a 50-km radius around the town of Lolodorf, in the south region of Cameroon (Supplemental Figure 1). This area was selected based on results from previous surveys, which demonstrated ICT prevalences of 7.7% (4/52) and 2.4% (1/42) in the villages of Melen and Bidjouka, respectively.^3^

### Study design and data collection (Figure 1).

#### Initial survey (May 2013): parasitological assessment of *L. loa* and *W. bancrofti* infection.

All individuals aged 5 years or older were invited to undergo parasitological examinations for filarial infection. Blood was collected by finger prick to detect CFA using the ICT (100 μL) read after 10 minutes according to the manufacturer's instructions. The result was reported using a semiquantitative score defined according to the relative intensity of test and control lines,^10^ that is, negative tests with no visible test (T) line were assigned a score of 0, tests with a T line weaker than the control (C) line were assigned a score of 1, tests with a T line approximately as dark as the C line were scored as 2, and cards with a T line darker than the C line were scored as 3.

**Figure 1.**
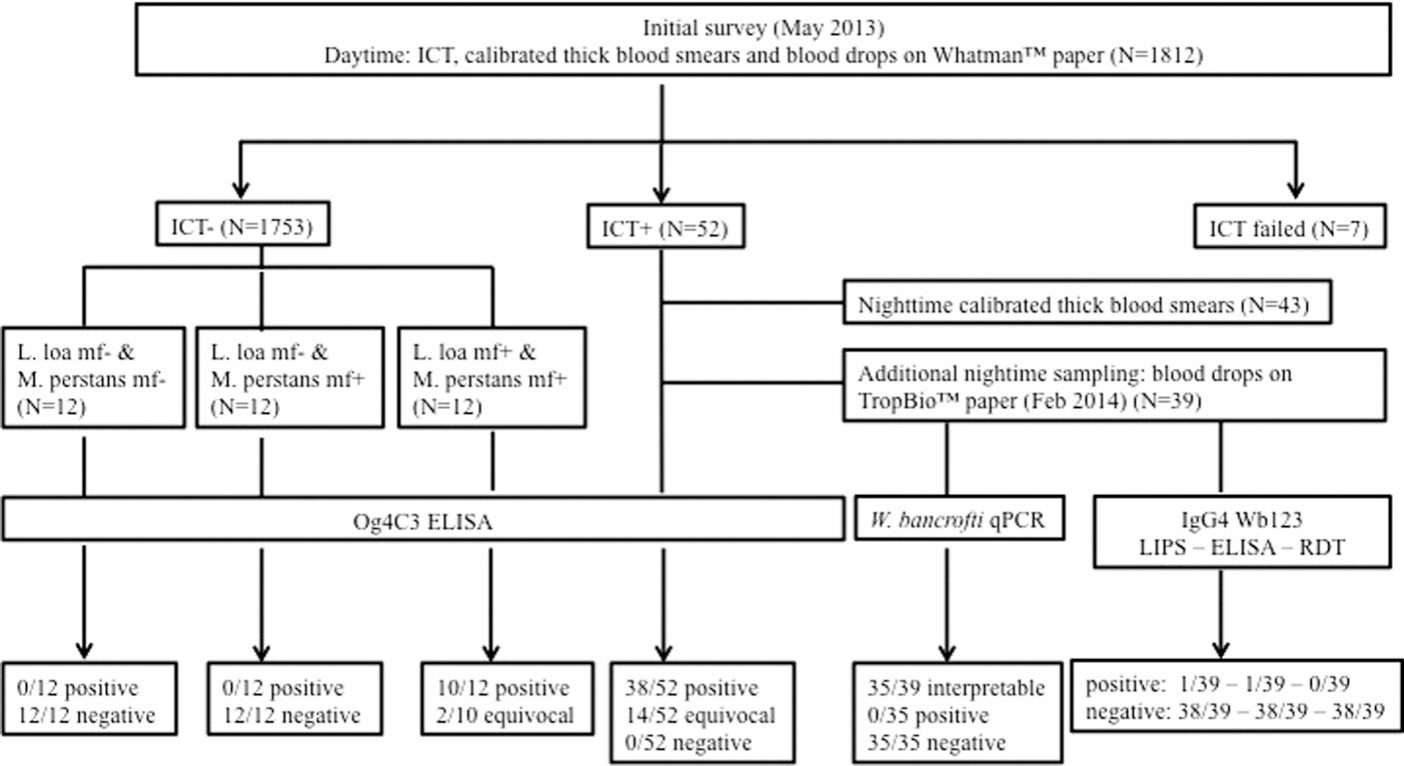
Data collection and main laboratory analysis results.

Standardized (50 μL collected with a microcapillary) thick blood smears were prepared to measure *L. loa* mf density. Another 50 μL of blood was collected on Whatman^®^ filter paper (GE Healthcare UK Ltd., Little Chalfont, United Kingdom) to quantify CFA levels using the TropBio^™^ Og4C3 Filariasis Antigen ELISA test (Cellabs Pty Ltd., New South Wales, Australia). Given the diurnal periodicity of *L. loa* microfilaremia, blood sampling was performed between 10:00 am and 4:00 pm. All individuals who were found positive by ICT were invited to be sampled again at night (after 10:00 pm) to prepare a thick blood smear (50 μL) to assess *W. bancrofti* microfilaremia.

All blood smears were stained with Giemsa within 48 hours and read by experienced microscopists. All mf present on the smear were counted. Mf of *Mansonella perstans*, also endemic in the study area, were distinguished from those of *L. loa* and *W. bancrofti* by size and the absence of a sheath.

#### Resampling of initially ICT-positive individuals (February 2014).

Previously identified ICT-positive individuals were resampled at night (after 10:00 pm) to provide appropriate material for extracting *W. bancrofti* DNA and for use in antibody assays. Thirty-nine of the original 52 ICT-positive individuals were able to be resampled (two died in the interval, 22 refused to be resampled, and nine were absent or had moved from their village). For each individual, 200–300 μL of blood was collected in an EDTA microtainer and dried blood spots (DBS) were prepared using TropBio^™^ filter papers (6–12 spots of 10 μL each). After drying, DBS were kept separately in individual plastic bags at ambient temperature during about 48 hours then were stored at −80°C until processing.

### Parasitological, molecular biology, and epidemiological analyses.

#### Detection of *W. bancrofti* and *L. loa* microfilarial DNA by species-specific qPCR.

DNA was extracted from 100 μL of whole blood collected using a DNeasy Kit (QIAGEN, Valencia, CA) and a final elution volume of 200 μL. qPCR assays for *W. bancrofti* were performed using 2 μL of DNA and the *W. bancrofti*-specific long DNA repeat (LDR) primers/probes previously described.^11^ All assays were performed in duplicate using the Kapa Probes Master Mix Kit (Kapa Biosystems, Wilmington, MA) with 20 pmol of each primer (LDR1 and LDR2) and 6 pmol of the LDR probe per well in a final volume of 20 μL and the FastPCR program (95°C 20 seconds, 40 cycles of 95°C 1 seconds and 60°C 20 seconds) in a StepOnePlus PCR system (Applied Biosystems, Foster City, CA). In addition, qPCR assays targeting the sequence LLMF72 were performed to identify DNA from *L. loa* mf^12^ in these night blood samples. These tests were performed using 1 μL of extracted DNA (representing 0.1427 μL of whole blood) for each qPCR using conditions described previously.^13^

#### Quantitation of CFA using the Og4C3 enzyme-linked immunosorbent assay test.

To further document the possible association between filarial antigenemia and *L. loa* mf levels, Og4C3 assays were performed using 50 μL DBS collected in May 2013 (initial survey) from the 52 ICT-positive individuals and from 36 selected ICT-negative individuals. The selection of the ICT-negative individuals was based on the *L. loa* and *M. perstans* mf densities found in their daytime blood smear. The 36 ICT-negative subjects were divided into three groups. The first group consisted of 12 individuals selected at random from the 958 people who were amicrofilaremic both for *L. loa* and *M. perstans*, who had never taken anthelmintic therapy and who had resided in the village during the prior 5 years. The second group consisted of the 12 subjects with the highest *L. loa* microfilarial levels among those who were amicrofilaremic for *M. perstans*. The third group consisted of the 12 subjects with the highest *M. perstans* microfilarial levels among those who were amicrofilaremic for *L. loa*.

For each sample, the test was performed in duplicate, and the results were expressed as the mean optical density (OD) of the two assays. The results were interpreted by comparing the OD of the sample with those obtained with the control standard samples included in the kit. The result was considered negative when the OD of the sample was lower than the OD of the control standard sample number 1 (corresponding to < 32 antigens unit [U/mL]), indeterminate when the OD of the sample was between the ODs of standard sample numbers 1 and 2 (32–128 U/mL), and positive when the OD was higher than the standard sample number 2 (group 3: 128–512 U/mL; group 4: 512–2,048 U/mL; and group 5: 2,048–8,192 U/mL).

#### Detection of IgG4 antibodies to Wb123.

Research of IgG4 antibodies to Wb123 were performed on eluted blood spots using three different immunoassays: 1) the luciferase immunoprecipitation systems (LIPS),^7^ 2) enzyme-linked immunosorbent assay (ELISA),^14^ and 3) prototypes of the Wb123/Ov16 biplex test.^14^

#### Statistical analyses—relationship between ICT positivity and *L. loa* infection.

Since the parasitological data were nonnormally distributed, unless otherwise stated, medians were used as the measure of central tendency. The relationships between ICT positivity and *L. loa* infection were assessed using data from the initial survey (May 2013). At the individual level, the association between ICT positivity and *L. loa* mf density was first explored using Cuzik's test for trend among the deciles of positive *L. loa* microfilarial densities. This association was then examined using a multivariate logistic regression model where the dependent variable was ICT status and the covariates were *L. loa* mf density, gender, age, and *M. perstans* mf density (continuous variable). A possible contextual effect of the community of residence was accounted for by including a village-level random effect in the model.

At the community level, the relationships between the rate of ICT positivity and the indicators of *L. loa* endemicity (prevalences and mean mf densities) were analyzed using pairwise correlations.

### Ethical agreement.

This study received ethical approval from the Cameroon National Ethics Committee (No. 002/CNERSH/SE/2012) and administrative approval from the Division for Health Operations Research at the Ministry of Public Health of Cameroon. The analyses performed at the National Institutes of Health (NIH) were performed on coded samples that were deemed exempt by the Office of Human Subjects Research.

## Results

### Prevalence and intensity of *L. loa*, *W. bancrofti*, and *M. perstans* microfilaremia, and ICT positivity.

During the initial survey (May 2013), a total of 1,812 persons aged 5 years and older were examined, including 969 females (53.5%) and 843 males (46.5%). The mean age of participants was 43.2 years and the median 44.0 years (interquartile range [IQR]: 21.5–62). The prevalence of *L. loa* and *M. perstans* microfilaremia in the entire sampled population was 23.4% (range among villages: 8.1–44.1%) and 36.1% (range among villages: 3.9–73.5%), respectively (Supplemental Table 1). ICT results were available for 1,805 subjects, of which 52 (2.9%) were found to be positive; four cards were scored at 3, nine at 2, and the remaining 39 at 1. ICT-positive individuals were found in 17 of the 26 communities surveyed. The proportion of ICT positivity ranged from 0% to 14.3% (the latter value was from a village where only 21 individuals could be examined). Among the 52 ICT-positive subjects, 43 agreed to be sampled again at night; none of them were infected with *W. bancrofti* mf but 39 individuals (90.7%) were found with *L. loa* microfilaremia (median: 1,240 mf/mL; IQR: 310–2,740; range: 20–18,540 mf/mL). *M. perstans* microfilaremia was observed in 19 (44.2%) of the 43 individuals, with median mf count of 300 mf/mL (IQR: 100–400; range: 20–9,960 mf/mL).

### Molecular biology analyses.

#### Detection of *W. bancrofti* mf DNA by qPCR.

qPCR for *W. bancrofti* and *L. loa* was interpretable for 35 of the 39 resampled subjects. All 35 samples were negative in the *W. bancrofti* LDR qPCR assay. The qPCR performed using the *L. loa*-specific LLMF72 sequence showed that there was no issue with DNA extraction in that 16 of the night blood samples were qPCR positive. Indeed, *L. loa* DNA was present and the relative amount of DNA based on cycle threshold values was positively associated with the density of *L. loa* mf observed in the night blood smears (Cuzik's test for trend, *P* < 0.0001) from the same blood samples.

#### Quantification of CFA using Og4C3 ELISA assay in ICT-positive individuals.

All 52 samples from the ICT-positive individuals were above 32 U/mL, the absolute negative cutoff for the assay. A total of 14 samples had equivocal results (levels between 32 and 128 U/mL). The remaining 38 samples (73.1%) were clear positive; 33 had levels between 128 and 512, four between 512 and 2,048, and one was above 2,048 U/mL. There was a positive relationship between antigen levels as measured by the Og4C3 ELISA and *L. loa* mf density (Cuzick's test for trend, *P* < 0.0001), and between the former and ICT score (Table 1, Cuzick's test for trend, *P* < 0.0001).

#### Quantification of CFA using Og4C3 ELISA assay in selected ICT negative individuals.

None of the 12 individuals without *L. loa* and *M. perstans* mf was positive in the Og4C3 ELISA assay. None of the 12 individuals harboring only *M. perstans* mf (range: 4,460–19,060 mf/mL) was positive in the assay. In the group of 12 individuals harboring only *L. loa* mf (range: 29,960–54,680 mf/mL), 10 (83.3%) had a positive ELISA result: nine in titer group 3 (128–512 U/mL), and one in group 4 (512–2,048 U/mL). The two other individuals were classified as indeterminate, with OD values higher than the highest value in individuals with no *L. loa* mf.

#### IgG4 antibodies to Wb123 in ICT-positive individuals.

Using the IgG4 anti-Wb123 LIPS assay, 1/39 sample was just above the cutoff for positivity. Using the IgG4 anti-Wb123 ELISA assay, 1/39 was also just above the cutoff for positivity but was different from the sample found positive by LIPS. Using the highly specific and standardized IgG4-based rapid diagnostic test (Wb123/Ov16 biplex test), all samples were negative. Thus, all 39 samples would be considered negative for IgG4 antibodies to Wb123 (based on negativity in at least two of three immunoassays).

### Epidemiological analyses.

#### Relationship between *L. loa* mf density and ICT results at the individual level.

Fifty (96.2%) of the 52 ICT-positive individuals had detectable *L. loa* mf in their daytime blood smears. The median *L. loa* microfilaremia in those 50 individuals was 24,330 mf/mL, (IQR: 10,775–43,365; range: 1,580–195,460 mf/mL).

In ICT-negative individuals, the proportion of *L. loa* mf carriers was 21.3% (373/1,753). Among the 373 microfilaremic ICT-negative individuals, median *L. loa* microfilaremia was 2,130 mf/mL (IQR: 380–7,945; range: 20–55,680 mf/mL). The *L. loa* mf densities in ICT-positive individuals were significantly higher than in ICT-negatives (Kolmogorov–Smirnov test, *P* < 0.001). From another perspective, the proportion of positive ICT increased gradually from the first to the 10th decile of positive *L. loa* microfilarial densities, exceeding 30% in subjects harboring more than 15,000 mf/mL, and 50% in those with > 20,000 mf/mL (Table 2) (Cuzick's test for trend *P* < 0.004). The results of the logistic regression model confirmed this exponential increase in the proportion of positive ICT with increasing *L. loa* microfilarial density (Table 2). The model also showed that gender, age, and *M. perstans* mf density were not associated with ICT positivity. The association between *L. loa* mf density and ICT was not dependent on the place of residence (nonsignificant random effect of village level). The distribution of the ICT score in relation to *L. loa* microfilaremia (presented in quartiles) is given in Table 3. All 13 subjects who had an ICT score of 2 or 3 are in the silo of the highest *L. loa* mf density (> 12,120 mf/mL). The eight subjects with the highest mf densities (≥ 77,880 mf/mL) were ICT positive; three had an ICT score of 3, two of 2, and three of 1.

#### Relationship between *L. loa* mf density and ICT results at the community level.

**Figure 2.**
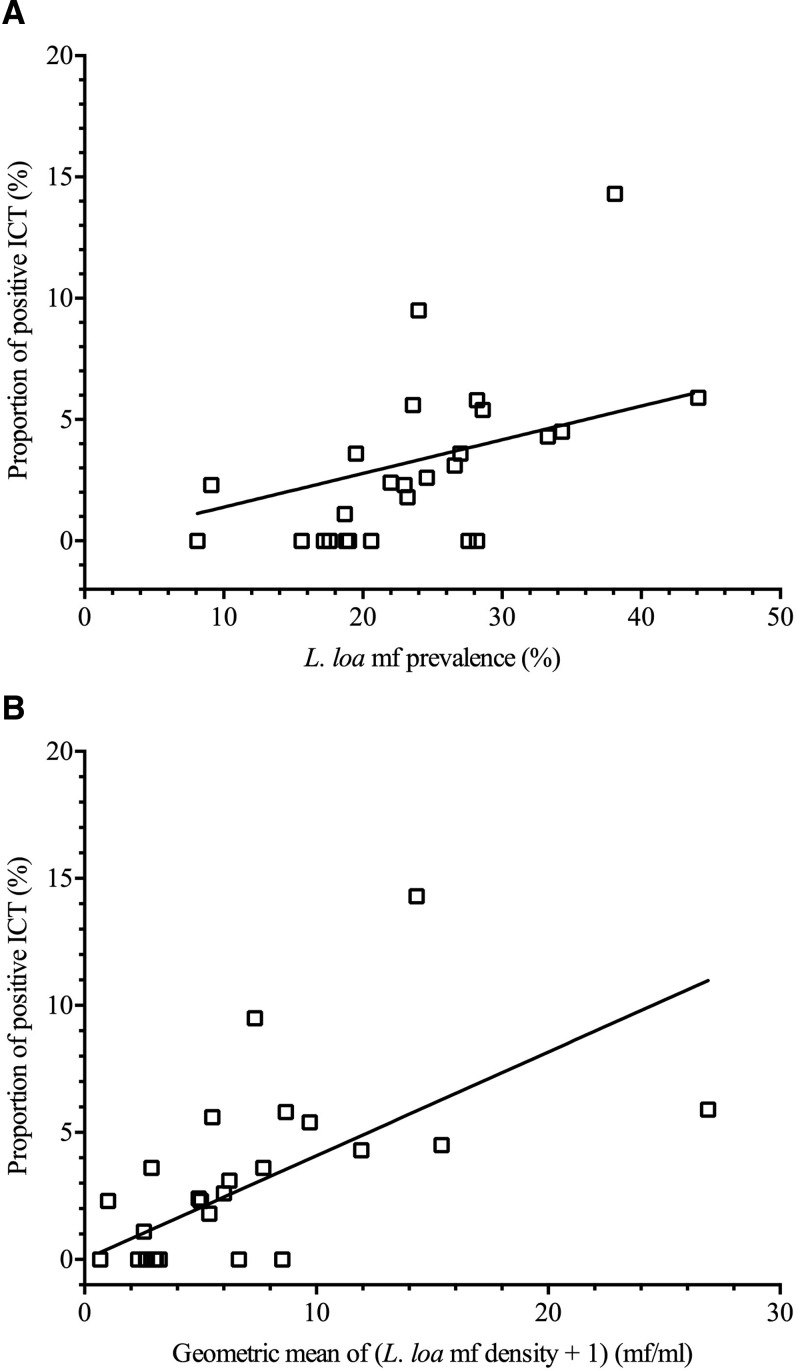
Relationship between the proportion of positive immunochromatographic card test (ICT), and (**A**) the prevalence of *Loa loa* microfilaremia (mf) and (**B**) the geometric mean of *L. loa* mf densities (mf/mL) in 26 villages of southern Cameroon. The lines represent the linear trend between the two variables.

There were significant positive correlations between the proportion of positive ICT and 1) the prevalence of *L. loa* mf (Pearson's *r* = 0.61; *P* = 0.001; Figure 2A

) and 2) the geometric mean of *L. loa* mf densities (Pearson's *r* = 0.57; *P* = 0.002; Figure 2B). These data suggest that, in most villages, the proportion of positive ICT would exceed 2% when the prevalence of *L. loa* mf in the total population is above 20% (Figure 2A).

## Discussion

For the past decade (or more), the rapid diagnostic ICT test for CFA has been the mainstay of mapping efforts for *W. bancrofti* and for surveillance following the cessation of mass drug administration (MDA). In most parts of the world, the utility of the ICT has been shown to be extraordinary. However, in areas of central Africa where *L. loa* is endemic and where the prevalence of ICT positivity was low, but not below the 1% threshold for triggering MDA, there appeared to be discrepancies between the results of the ICT and those from night blood smears for *W. bancrofti* mf. Indeed, in two recent studies (performed concurrent to the present study), CFA positivity appeared to be associated with high levels of *L. loa* mf in the blood.^4^,^5^ The present study adds to these studies by examining in a new area the association, at both the community and individual level, between *L. loa* mf and CFA and, most importantly, by using additional qualitative and quantitative diagnostic tools to demonstrate the relationship between high grade *L. loa* microfilaremia and filarial antigenemia.

As a backdrop to the present study, the prevalence of circulating filarial antigenemia in the Lolodorf health district (south Cameroon) was found to be 2.9%. In this area, *L. loa* and *M. perstans* filariasis are highly endemic, providing an ideal location for examining this interaction. Although suspecting that the ICT positivity was caused by a possible cross-reaction between *L. loa* and *W. bancrofti*, we applied all available methods to confirm the absence of *W. bancrofti* infection in the ICT-positive individuals. Using these methods (Og4C3 ELISA, IgG4 antibodies to Wb123), we definitively demonstrate that *L. loa* infection associated with high microfilaremia in the absence of true *W. bancrofti* infection can lead to false-positive tests for *W. bancrofti* CFAs. Moreover, given the relatively low threshold (ICT positivity > 1%) for implementing MDA for LF, even a small degree of cross-species reactivity can make an area eligible for a LF control program.

Multivariate analyses demonstrated a strong association between high daytime *L. loa* mf levels and the ICT results (qualitative and semiquantitative) and the quantitative CFA levels using the Og4C3 ELISA. In the same analyses, *M. perstans* mf density was not associated with ICT result, possibly indicating that *M. perstans* adult worms do not produce cross-reactive CFA (see next paragraph). Another explanation could be that *M. perstans* adult worms and their antigens are contained within the serous membranes that confine their anatomical location.^6^

The overwhelmingly likely explanation for the present findings is that the CFA detected by both the ICT and the Og4C3 ELISA is also produced by *L. loa* (and potentially by the other pathogenic filariae such as *Onchocerca volvulus* and *Brugia malayi*). This might well be the case because the monoclonal antibody used in the ICT test (AD12) was originally produced by immunizing mice with antigens from the dog heartworm *Dirofilaria immitis*,^15^,^16^ and the Og4C3 monoclonal antibody was induced by raising antibodies to male *Onchocerca gibsoni*. Both of these antibodies have been shown to bind to other filarial species using immunoblot analysis.^17^ A recent study showed that mf and L3s of *L. loa* maintained in vitro generate excretory/secretory (E/S) products that induce positivity of ICT test.^6^ In this study, the same result was also obtained using E/S products from *Onchocerca ochengi* adult males maintained in vitro. The same investigators also showed, using in vivo systems, that sera and whole blood from baboons experimentally infected with *L. loa* L3s induced ICT positivity, but that sera and whole blood from cattle naturally infected with *O. ochengi* did not cause ICT positivity.^6^

Actually, very little information is available on the specificity of the ICT, the AD12-based antigen ELISA assay, or the Og4C3 ELISA for the diagnosis of *W. bancrofti* infection in humans. In one small study, one of 12 patients infected with *O. volvulus* but none of four with *L. loa* mf were positive in an AD12-based ELISA.^16^ In a second study, Og4C3 ELISA assay performed using a pool of sera from seven patients infected with *L. loa* was found to be negative.^17^ A third small study demonstrated false-positive ICT results in 2/40 subjects with dracunculiasis, one of whom being also positive by the Og4C3 ELISA.^18^

The complete negativity of the sera from the present study in the Wb123/Ov16 biplex IgG4-based assay and the relative negativity (38/39) in the IgG4-based WB123 LIPS and ELISA assays provide more compelling evidence of *L. loa*-induced false ICT positivity. As reported previously, IgG-based Wb123 assays are a sensitive and ∼95–98% specific marker of exposure to *W. bancrofti* infection.^7^,^9^ Of note, during the initial development of the IgG Wb123 LIPS assay in the NIH laboratory, using samples different from those collected as part of the present study, the test was positive in only one of 62 subjects with *L. loa* infection.^7^ Although the presence of antibodies to Wb123 does not distinguish between active and past infection, the negative results provide additional evidence that no *W. bancrofti* infection was present in our study subjects.

Among the 52 subjects with a positive ICT, only two had no *L. loa* mf. Those two individuals had settled in the villages where they were examined 11 months and 4 years before the survey. They both previously lived in areas where loiasis was also endemic and were thus probably both harboring *L. loa* adult worms. Absence of *L. loa* mf may indicate that they had single sex infection, or that they were genetically not predisposed to microfilaremia.^19^ Although they both stated that they had not taken any antifilarial treatment before the survey, we cannot be completely assured that they did take a microfilaricidal drug for *L. loa* before the survey.

Our data, which definitely demonstrate positive CFA in the setting of high *L. loa* infection, have significant implications in terms of mapping and control of LF caused by *W. bancrofti* in *Loa*-endemic areas. The extent of this issue depends on the actual population living in loiasis/LF co-endemic areas and is, for the time being, a matter of speculation.^2^ Besides, it should be noted that our results do not suggest that ICT would be of no use in *Loa*-endemic areas. ICT testing could still be used to identify regions that do not require MDA for LF (the latter being defined as those where the antigenemia rates, as assessed by the ICT, or the microfilaremia rates for *L. loa* are strictly < 1%).^20^ Conversely, when the ICT prevalence is equal to or greater than 1% and *L. loa* is present, other options should be considered. One option would be to do no further testing and to proceed as if *W. bancrofti* transmission is ongoing, that is, to organize MDA using 6-monthly treatment with albendazole alone (if possible associated with mosquito control), as currently recommended by the World Health Organization.^21^ This would likely provide MDA to fairly large areas where LF is not endemic, but there would be a beneficial impact on the community through control of soil-transmitted helminth infections, which are prevalent throughout central Africa. A second option would be to definitively confirm *W. bancrofti* endemicity before launching MDA against LF through the use of a second test (microscopy, PCR, or the newly launched Wb123-based mono- or biplex test) in ICT-positive individuals. An upcoming large-scale field trial employing the Wb123/Ov16 biplex to assess onchocerciasis prevalence in two *Loa*-endemic areas in Cameroon that are not endemic for LF will provide definitive information on the utility of Wb123 test to define *W. bancrofti* endemic areas in central Africa. Of note, this test would be much easier to apply than the other methods (microscopy or PCR) that require sampling night blood.

Given that *L. loa* infections with high levels of microfilaremia lead to positive ICTs, there is concern that the launching of the more sensitive Alere Filariasis Test Strip (Alere Inc.)^22^ could lead to a significant increase in CFA rates in *Loa*-endemic regions. As MDA efforts for LF are being rolled out in many of these central African countries, evidenced-based policies need to be provided to the program managers to ensure success of these very important elimination programs.

## Supplementary Material

Supplemental Datas.

## Figures and Tables

**Table 1:** Relationship between the ICT score and the antigen levels as measured by the Og4C3 ELISA in 52 ICT-positive subjects

ICT score*	Og4C3 titer group (antigens unit)					Total
	0–32 (negative)	32–128 (equivocal)	128–512	512–2,048	> 2,048	
1	0	13	25	1	0	39
2	0	1	8	0	0	9
3	0	0	0	3	1	4
Total	0	14	33	4	1	52

ELISA = enzyme-linked immunosorbent assay; ICT = immunochromatographic card test.

* 0 = no test (T) line; 1 = T line thinner than control (C) line; 2 = T line as thick as C line; 3 = T line thicker than C line.^10^

**Table 2:** Results of the logistic regression model assessing the factors associated with ICT positivity

Variable		ICT positivity* (%)	OR	95% CI	*P* value
*Loa loa* density (mf/mL)	≥ 29,460–195,460	22/42 (52.4)	639.3	147.7–2,768.0	< 0.0001
	15,380–29,220	13/42 (31.0)	271.5	76.1–969.5	< 0.0001
	8,820–15,100	5/43 (11.6)	75.7	13.4–427.7	< 0.0001
	5,180–8,700	6/42 (14.3)	93.8	18.8–467.5	< 0.0001
	3,060–5,160	3/43 (7.0)	43.8	7.4–259.5	< 0.0001
	1,540–3,040	1/42 (2.4)	15.4	1.3–188.1	< 0.0001
	740–1,480	0/41 (0)	1 (empty)		
	300–720	0/43 (0)	1 (empty)		
	140–280	0/40 (0)	1 (empty)		
	20–120	0/45 (0)	1 (empty)		
	0	2/1,753 (0.1)	1 (reference)		
Age	Continuous		1.0	0.9	0.507
Sex	Female		1 (reference)		
	Male		1.6	0.8	0.171
*Mansonella perstans* density	Continuous		1.0	0.9	0.870
Constant			0.0009		< 0.001

CI = confidence interval; ICT = immunochromatographic card test; OR = odds ratio.

* ICT positivity rate in the 10 *L. loa* microfilarial density deciles and in amicrofilaremic subjects, unadjusted on other covariates.

**Table 3:** Relationship between the ICT score and the *Loa loa* mf (distributed in quartiles)

ICT score*	*L. loa* mf density (mf/mL)					Total
	0	1–480	481–3,060	3,061–12,120	> 12,120	
0	1,380	110	101	93	69	1,753
1	2	0	1	13	23	39
2	0	0	0	0	9	9
3	0	0	0	0	4	4
Total	1,382	110	102	106	105	1,805

ICT = immunochromatographic card test; mf = microfilaremia.

* 0 = no test (T) line; 1 = T line thinner than control (C) line; 2 = T line as thick as C line; 3 = T line thicker than C line.^10^
