# Supplementary material for: Positivity of Antigen Tests Used for Diagnosis of Lymphatic Filariasis in Individuals Without Wuchereria bancrofti Infection But with High Loa loa Microfilaremia
Source: Am J Trop Med Hyg. 2016 Dec 7;95(6):1417–23. doi: 10.4269/ajtmh.16-0547 (PMC5154460; doi:10.4269/ajtmh.16-0547)
Supplement: Supplementary file 1 [file SD7.pdf]

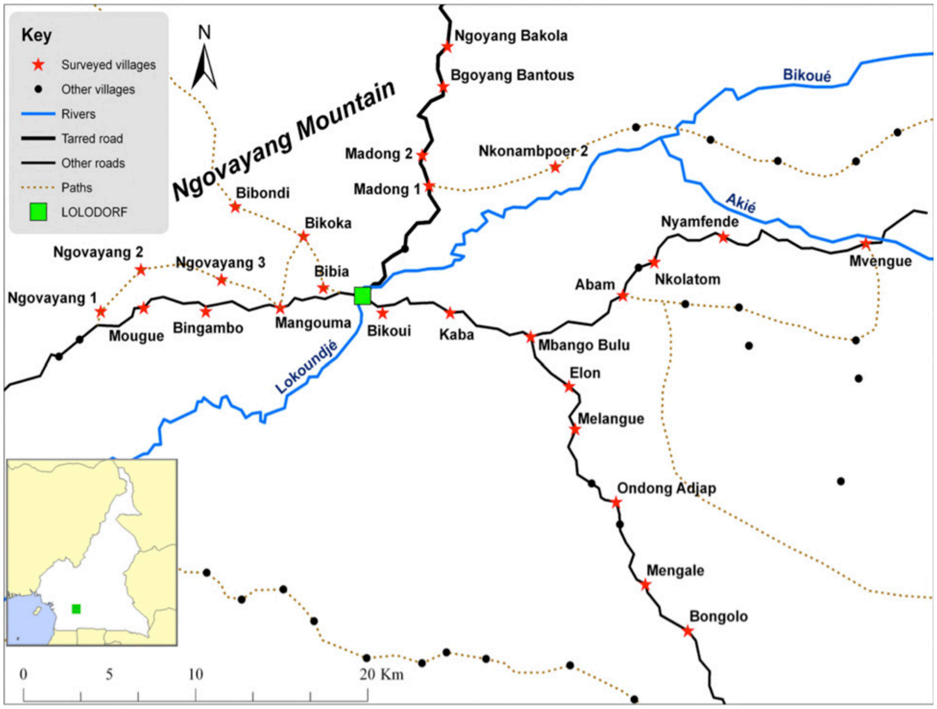

Supplemental Figure 1. Location of the villages surveyed in the Lolodorf study area.

Supplemental Table 1

Prevalences of *Loa loa* and *Mp* mf and proportion of positive ICT in the 26 surveyed communities

Village	Latitude	Longitude	No. examined	% *Loa* mf	% *Mp* mf	% ICT+
Nkolatom	3.26304	10.86057	21	38.1	52.4	14.3
Mangouma	3.22466	10.78517	75	24.0	49.3	9.5
Bingambo	3.2301	10.66123	68	44.1	66.2	5.9
Odong Adjap	3.14084	10.84687	103	28.2	3.9	5.8
Ngovayang II	3.2484	10.64158	72	23.6	50.0	5.6
Ngoyang Bantous	3.34496	10.75742	56	28.6	62.5	5.4
Ngovayang I	3.22822	10.60726	67	34.3	59.7	4.5
Bongolo	3.07905	10.88036	48	33.3	25.0	4.3
Mvengue	3.28306	11.16667	113	19.5	4.4	3.6
Nyamfende	3.2788	10.89074	111	27.0	17.1	3.6
Elon	3.2007	10.82183	64	26.6	20.3	3.1
Bikoka	3.26869	10.6911	114	24.6	27.2	2.6
Ngovayang III	3.24435	10.65049	41	22.0	58.5	2.4
Nkonambpoer 2	3.23352	10.72774	87	23.0	56.3	2.3
Bikoui	3.25	10.71667	88	9.1	25.0	2.3
Mengale	3.09761	10.86609	56	23.2	19.6	1.8
Mbango Boulou	3.21606	10.807	91	18.7	18.7	1.1
Bibia	3.23439	10.69397	48	18.8	25.0	0
Bibondi	3.27999	10.66078	71	28.2	43.7	0
Mougue	3.21608	10.61283	34	20.6	73.5	0
Ngoyang Bakola	3.35859	10.75532	74	8.1	55.4	0
Madong I	3.28489	10.75778	105	19.0	50.5	0
Madong II	3.27743	10.75628	32	15.6	65.6	0
Abam	3.18022	10.82507	58	27.6	32.8	0
Melangue II	3.17989	10.82657	64	17.2	23.4	0
Kaba	3.2788	10.89074	51	17.6	51.0	0
Total			1,812	23.4	36.1	2.9

Mf = microfilaremia; Mp = *Mansonella perstans*.
